# Matching the pitch perception of the cochlear implanted ear with the contralateral ear in patients with single-sided deafness: a novel approach

**DOI:** 10.1007/s00405-023-08002-z

**Published:** 2023-05-03

**Authors:** Tamás Ferenc Tóth, Adrienne Németh, Péter Bakó, Péter Révész, Imre Gerlinger, István Szanyi

**Affiliations:** Department of Otorhinolaryngology and Head Neck Surgery, Medical School, University of Pécs, 2. Munkácsy M. Str., Pécs, 7621 Hungary

**Keywords:** Auditory adaptation, Cochlear implant, Interaural pitch-matching, Single-sided deafness

## Abstract

**Purpose:**

Single-sided deaf patients following cochlear implantation often compare the sound quality of their implanted ear with normal hearing. The interaural differences can result in dissatisfaction with speech comprehension and reduced time of usage of the speech processor; hence, prolonging auditory adaptation time. The proposed calibration method presented in this study demonstrates how the frequency distribution of the cochlear implant can be set to adequately approximate the pitch perception of the contralateral normal hearing ear towards improving speech intelligibility in a noisy environment.

**Methods:**

In 12 postlingual single-sided deaf patients, subjective interaural pitch-matching was carried out to determine new central frequencies for the reallocation of the frequency bands of their speech processor (CP910, CP950 or CP1000, Cochlear, Australia). The patients were asked to compare the pitch of the tones presented to their normal hearing ear to the pitch of individual channels of their cochlear implant (CI522 or CI622, Cochlear, Australia). A third-degree polynomial curve was fit to the acquired matching frequencies to create the new frequency allocation table. Audiological measurements (free-field aided thresholds, speech reception thresholds, and monosyllabic word recognition score) in noise, together with a Speech, Spatial and Qualities of Hearing Scale (SSQ12) questionnaire (short version of the original SSQ) results were evaluated prior to the pitch-matching procedure, and again, 2 weeks later.

**Results:**

The free-field aided thresholds of the patients showed no greater shift than ± 5 dB following the procedure; however, their monosyllabic word recognition score in noise improved significantly (mean − 9.58%, SD 4.98%, matched pairs *t* test comparison: *p < *0.001). The results of the SSQ12 questionnaire also showed significant improvement in speech intelligibility, sound localization, and sound quality (mean 0.96 points, SD 0.45 points, matched pairs *t* test comparison: *p < *0.001).

**Conclusions:**

Matching the pitch perception of the implanted cochlea with the sensation of the normal hearing contralateral ear, resulted in significant changes in the quality of hearing in patients with single-sided deafness. It is plausible the procedure can usher positive results in bimodal patients or following sequential bilateral cochlear implantation.

## Introduction

The adequate means to rehabilitate single-sided deafness (SSD) has been a matter of debate addressed by comparative studies and systematic reviews in the recent past [1–3]. Many patients afflicted with SSD experience difficulties localizing sound sources, understanding speech in noisy environments, and enjoying concerts or live music [4–9]. Without the head-shadow effect [10, 11], the binaural squelch [12, 13], and the binaural redundancy effect [14, 15], everyday listening situations can prove exhausting for both children and adults. In numerous cases, patients also complain about mild to moderate tinnitus in the affected ear, impairing their general quality of life [6, 16].

The first standard recommendation for the treatment of SSD was to transfer the sound from the deaf ear’s side into the normal hearing cochlea via the Contralateral Routing of Signals (CROS) or with the application of a Bone Conduction Device (BCD), e.g., Bonebridge or BAHA [17, 18]. CROS systems and BCD solutions improve speech understanding of SSD patients, mostly in less noisy environments. Nevertheless, they are proven ineffective in sound source localization; therefore, they do not increase the level of speech comprehension in background noise, especially in commonly referred to, “cocktail party” situations, where the brain must filter out a wide range of stimuli similar to the conversation being focused on [7].

Cochlear implantation (CI) aims to rehabilitate the hearing of the impaired ear rather than transferring information to the contralateral side, restoring the patient’s access to binaural cues. CI has been demonstrated to improve sound localization and speech comprehension in noisy environments and significantly reduce tinnitus severity in adult SSD patients [6, 16, 19].

As medical technology advances and newly published studies surface, the indication criteria for specific implantable hearing-aid devices often experience minor—or sometimes even significant—changes. In July 2019, the US Food and Drug Administration (FDA) first approved MED-EL cochlear implantation for single-sided deaf children 5 years or older [20]. Today, if financially accessible, it is also the preferred choice for rehabilitating SSD in adults.

The perceived pitch of a tone is a function of not only the place of stimulation in the cochlea (tonotopy) but also the stimulation rate of the vibration. Moreover, the mismatch between the frequency and place encoding may negatively affect auditory processing. It has been demonstrated by Rader et al. that using place-dependent stimulation rates in CI improves tonotopic pitch perception in patients with SSD [21].

Applying image-guided shifting or modification of the frequency allocation to match the individual tonotopy of cochlear implantees may also result in better pitch perception [22]. Grasmeder compared audiological results of different frequency mapping techniques based on the Greenwood function, the Spiral Ganglion (SG) frequency-position function, a reduced frequency range map, and the original clinical maps of the participants and found significant differences regarding vowel and sentence perception [23]. Greenwood’s equation [24] considers the frequency distribution along the Organ of Corti (OC); nevertheless, CI electrodes stimulate neurons in the SG. As a form of correction, the frequency-position function of the SG has been introduced by Stakhovskaya [25].

The considerations and techniques referred to above are convenient if a High-Resolution Computed Tomography (HRCT) scan of the patient’s cochlea is available for image-guided mapping, such as MED-EL’s anatomy-based fitting provided by the connected application of the OTOPLAN and MAESTRO software [26–30]. However, even with a frequency band reallocation following the best estimations based on the position of the electrode contacts in the cochlea, it is not evident SSD patients stimulated with a given frequency sound will sense the same pitch in both ears.

Involving the patients in optimizing their frequency maps and utilizing the guidance of their subjective pitch sensation may serve as a valuable tool in the hands of the audiologist. SSD patients and bimodally aided CI users can serve as ideal candidates for the pitch-matching procedure due to their motivation to minimize disturbing interaural frequency mismatch; hence, acquiring more natural bilateral sonority of hearing and achieving better speech comprehension, especially in noisy environments.

## Materials and methods

### Patient selection

Twelve patients (6 male, 6 female) with acquired postlingual single-sided deafness (pure-tone air conduction thresholds ≤ 25 dB HL in the non-implanted ear) were included in the current study (Table 1). The patient group demonstrated heterogeneity in their age at implantation (mean 42 years, SD 16.75 years). All participants were previously implanted with CI522 or CI622 (Cochlear, Australia) cochlear implants and fitted with Nucleus™ CP910, CP950, or CP1000 speech processors (Cochlear, Australia). All subjects were experienced CI users (1–5 years of daily usage of 8–17 h) with stable audiological results. Although they were agile listeners who can differentiate between the pitches of all 22 stimulating channels, they were not satisfied with the sonority and general tone of their devices nor the resulting poor speech comprehension in noise, especially when experiencing chattering speech-noise. All participants had been chosen specifically for this experiment to help them gain better tonal sonority of the CI, since no other fitting methods (e.g., changing to Neuro-Response Telemetry (NRT)-based mapping, changing coding strategy from ACE to MP3000, SPEAK or CIS, lowering stimulation rate or number of maxima) provided satisfying results. Our study was approved by the local Scientific and Research Ethics Committee of the Medical Research Council (Approvement number: 9398–2022).

**Table 1:** Demographic data of the participants

ID	Age (years)	Gender	Duration of deafness (years)	Implant usage (years)	Implant type	Processor type
1	26	Male	21	5	CI522 Slim Straight	CP950 Kanso
2	56	Male	5	4	CI522 Slim Straight	CP910 Nucleus6
3	45	Female	18	4	CI532 Slim Modiolar	CP910 Nucleus6
4	18	Female	15	3	CI522 Slim Straight	CP950 Kanso
5	49	Male	12	3	CI522 Slim Straight	CP950 Kanso
6	51	Female	6	3	CI522 Slim Straight	CP950 Kanso
7	25	Male	20	3	CI522 Slim Straight	CP910 Nucleus6
8	42	Female	0	2	CI522 Slim Straight	CP950 Kanso
9	53	Female	6	2	CI522 Slim Straight	CP950 Kanso
10	70	Male	2	2	CI522 Slim Straight	CP950 Kanso
11	17	Male	1	1	CI522 Slim Straight	CP1000 Nucleus7
12	52	Female	49	1	CI622 Slim Straight	CP950 Kanso

### Audiological measurements

All audiological measurements were carried out in a free sound field using a clinical audiometer (Piano, Inventis). The signal source loudspeaker was placed one meter lateral to the implanted ear of the patient, while a secondary loudspeaker produced a noise pattern (specified below for each measurement) one meter lateral to the contralateral ear, to simulate a noisy environment with the noise originating from the direction of the normal hearing ear. Free-field aided Pure-Tone Audiometry (PTA_4_) was performed, averaging the four main speech frequencies (0.5, 1, 2, and 4 kHz). Frequency-specific narrow band 60 dB SPL noise was presented by the secondary loudspeaker towards the normal hearing ear. Speech Reception Threshold (SRT_50%_) using sets of two-digit numbers, and monosyllabic Word Recognition Score (WRS_65dB_) were both tested using the official Hungarian tests [31] while presenting 60 dB SPL speech noise to the contralateral ear by the secondary loudspeaker. This setup aimed to mimic an everyday hearing situation in which both the signal and the noise could be heard with the contralateral ear also, yet not as loud as with the ipsilateral ear.

Patients were tested immediately prior to the fitting session and again, 2 weeks later, following the acclimatization to the new map. The pre-fitting measurement of each patient served as a personal baseline for future comparison.

### Questionnaire

The Speech, Spatial and Qualities of Hearing Scale (SSQ12) questionnaire was carried out in parallel to the audiological measurements in support of the evaluation of changes in patients’ speech intelligibility in complex listening situations and their sound localization abilities. SSQ12 is a short, validated version of the original Speech, Spatial and Qualities of Hearing Scale suitable for clinical use [32]. The twelve questions span three dimensions: speech comprehension in everyday noisy environments, sound localization skills, and sound quality and sonority regarding aided hearing. The patient can respond to each question by annotating a score on a scale, ranging from 0 (not at all) to 10 (perfectly) or marking the “not applicable” option. Several patients also provided more detailed information in reference to their personal experiences for specific questions and topics.

### Pitch-matching method

**Fig. 1:**
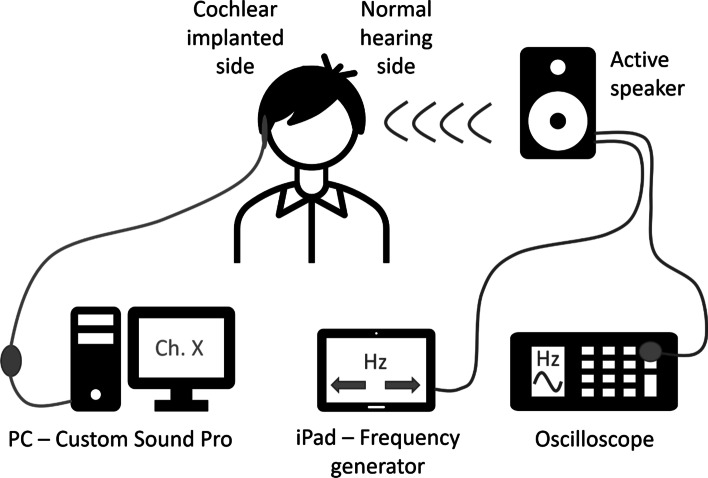
Interaural pitch-matching setup

An Apple iPad Air3 with a tone generator application (Audio Function Generator, Thomas Gruber) was used to produce the waveforms, and the acoustic stimuli were presented to the normal hearing side of the patient by an active loudspeaker (JBL One Series 104). Adel et al. demonstrated the choice of acoustic stimulus type can have a significant effect on electric–acoustic pitch-matching results [33]; therefore, different waveforms (sinusoid, rectangular, triangular, and sawtooth) were tested prior to the test. Based on the patient’s subjective discernment, the most similar one to the CI stimulation was selected for future measurements. The exact frequency of the generated tone was monitored in real-time using a digital oscilloscope (Owon SDS 1102). At the same time, a stimulus was presented through a specific channel of the cochlear implant system, in which the patient was asked to compare the pitch of the tones perceived at the two sides (Fig. 1).

**Fig. 2:**
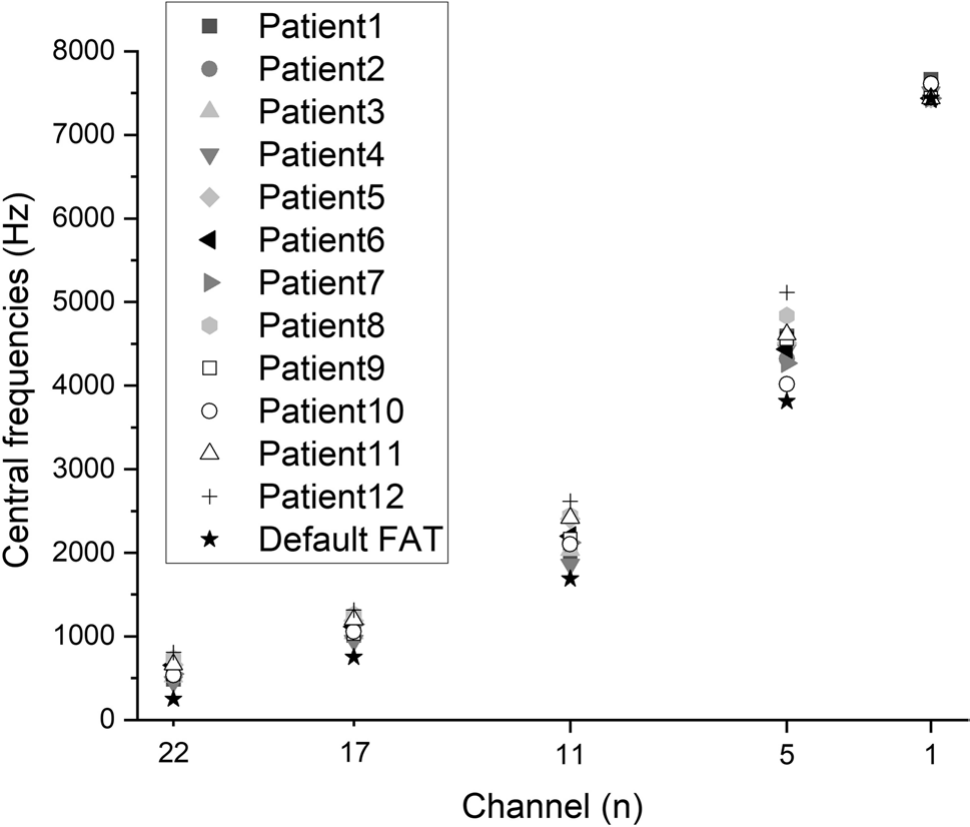
Measured central frequencies of the individual patients compared to the values of the default FAT

**Fig. 3:**
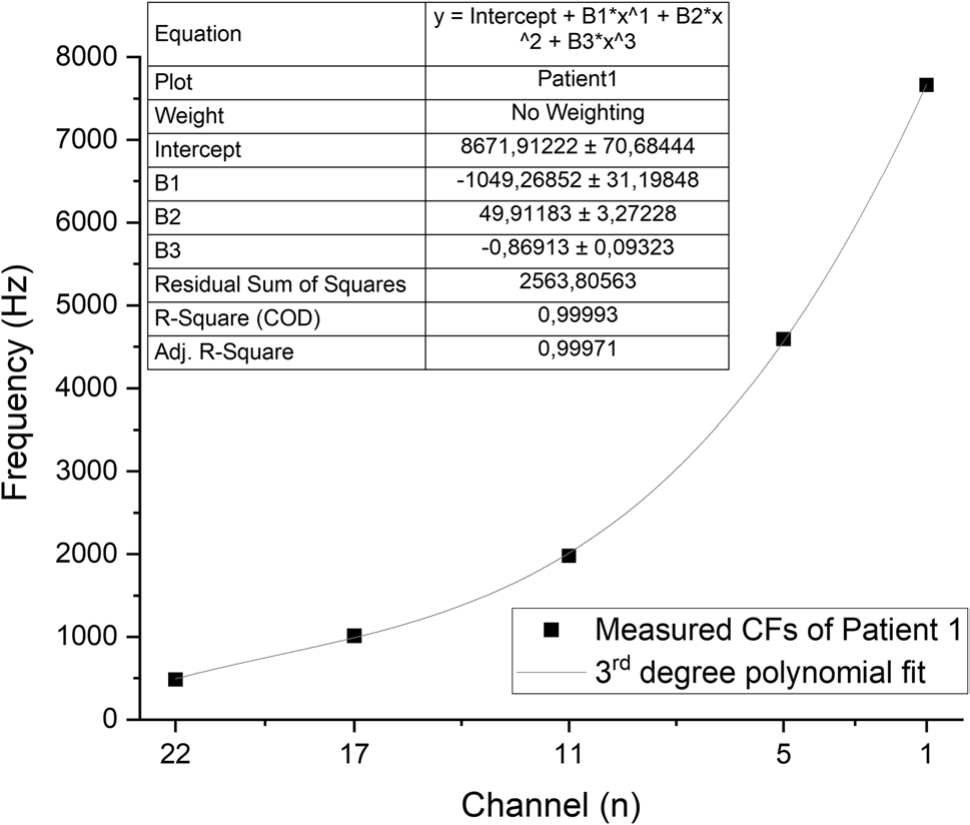
Example of third-degree polynomial curve fitting to the measured central frequencies (CFs)

**Fig. 4:**
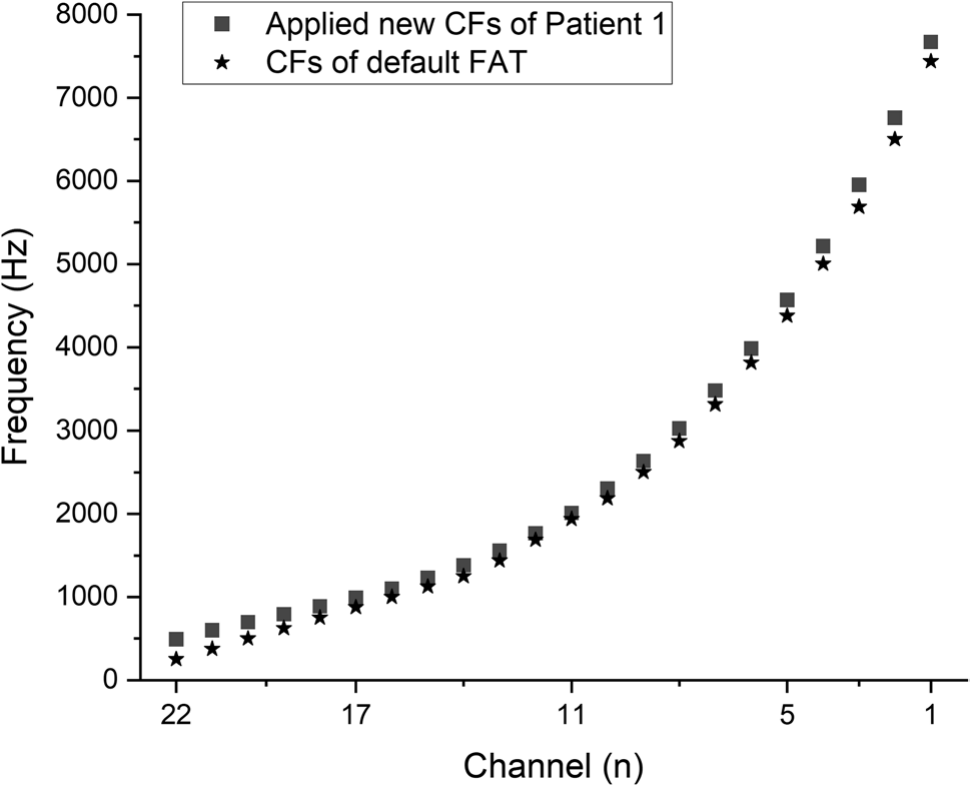
Example of frequency allocation table (FAT) generation

The tested CI channel was activated in Custom Sound Pro 6.3 (Cochlear, Australia) in 2 Hz intermittent presentation of pulse trains (rate: 900 pps, stimulus duration: 250 ms, inter-stimulus duration: 250 ms) at a relatively loud intensity level (the patient’s everyday threshold level (T-level) + 75% of the dynamic range of that individual channel). The volume of the acoustic tone produced by the loudspeaker on the contralateral side was adjusted to the perceived level of the electric stimuli of the CI based on the subjective discernment of the patient. Afterwards, the pitch of the continuous acoustic tone was adjusted by the audiologist to approximate the perceived pitch of the electric stimulus relying on the patient’s feedback. We ensured all patients understood their task during the pitch-matching procedure which specifically, was comparing pitches and not volume levels. Seamless ascending and descending approximations were carried out randomly between 0.1 and 20 kHz (also manually randomizing the speed of frequency adjustment) at least 4–5 times to determine the channel’s average Central Frequency (CF). All patients were asked to keep their eyes closed during the procedure to lower the bias of their subjectivity. Channels number 11, 17, 5, 22 and 1 were tested consecutively in the above-mentioned manner (Table 2). Figure 2 compares the measured CFs of individual patients with the factory default values. The CF of the highest frequency band (channel 1) often exceeded the limit (7938 Hz) of the CI system; therefore, in these cases, it was set to default (7438 Hz). Following the conclusion of the test above rather than merely interpolating the channels in between, a third-degree polynomial curve was fit to the acquired data set to determine CF values for the new Frequency Allocation Table (FAT), as demonstrated in Fig. 3. To form the frequency bands for the new FAT, the cutoff frequencies were set equidistantly from the neighboring CFs (Fig. 4). (In patient groups aided with 12 or 16 channel electrodes in consideration of the nonlinearity of the pitch perception Carlyon et al. applied geometric mean [34]).

**Table 2:** New CFs (Hz) of the individual participants (P1–12) compared to the default FAT values of the measured channels

CH	P1	P2	P3	P4	P5	P6	P7	P8	P9	P10	P11	P12	Default fat
22	484	537.25	520.5	455.25	658.75	655.25	553	734.75	544.5	533.75	664.25	807.25	250.5
17	1012	1063	1034	948.25	1185.5	1117.5	1146.5	1259	1030.25	1056	1202.5	1316	750.5
11	1978.75	2123.25	2033.25	1861.25	2404	2200.5	2122.75	2441.5	2164.75	2102	2414.5	2617.25	1688
5	4592	4319.5	4540.25	4428	4506.25	4434.5	4269	4835.5	4558.5	4017.5	4612.5	5114.25	3813
1	7661.75	7438^a^	7492.5	7510.75	7438^a^	7438^a^	7438^a^	7438^a^	7438^a^	7612.5	7438^a^	7438^a^	7438

^a^The measured CF exceeded the limit of the CI system (7938 Hz); therefore, it was set to the default CF value of channel 1 (7438 Hz)

To eliminate bias in the evaluation of the audiological results, none of the other parameters, e.g., T-levels (thresholds), C-levels (comfort levels), gains, repetition rate, number of maxima, loudness growth factor or coding strategy (ACE) were adjusted during the pitch-matching session. In consideration of the statistical analysis, a matched pairs *t *test comparison was applied in the operational use of the SPSS19 software.

## Results

The interactive portion of the procedure spanned an average of 20–30 min depending on how quickly the patient could accurately determine the acoustic tones matched with the five tested channels of the cochlear implant. The calculations and programming (creating the new map with the reallocated FAT) required approximately an extra 5 min—except in the first case, where an additional 15–20 min were required to create the template (OriginPro 2022, OriginLab Corporation) used to calculate the average CF values for each tested channels, to fit the third-degree polynomial curve to the acquired datapoints, and to calculate the lowest and highest frequency of each frequency bands for the new FAT.

Following a switch to the new map, nearly all the patients reported an immediate positive effect regarding their hearing quality and experienced a “more natural sounding” or described “more similar hearing to normal”. All patients were instructed to use only the new map for 2 weeks of testing. Changing the volume levels and the sensitivity of the microphones (if enabled) were allowed during the test period; however, both levels were set to default during the follow-up audiological measurements.

The summary of the audiological measurements and average scores of the SSQ12 questionnaire are presented in Table 3. The pre-fitting and post-fitting PTA_4_ measurements showed no difference (mean − 0.31 dB, SD 2.97 dB). This result substantiates, changing the FAT does not affect the hearing thresholds of the patients if the T-levels were correctly set prior to the procedure. The SRT_50%_ measurements demonstrated a similar outcome: the FAT rearrangement resulted in an average of 0.21 dB difference (SD 3.71 dB). However, the WRS_65dB_ demonstrated a significant improvement with the experimental map (mean − 9.58%, SD 4.98%, matched pairs *t *test comparison: *p < *0.001). The purpose of the SRT_50%_ test is to measure the minimal loudness level the patient requires for gaining sufficient vocal information to decide which phrase was presented from a pool. Therefore, this method might be less sensitive to interaural frequency mismatch and distortions than WRS_65dB_ in which the patient needs to clearly understand monosyllabic words without further clues. This may serve as an explanation to why WRS_65dB_ still improved despite potential ceiling effects while SRT_50%_ did not.

**Table 3:** Audiological (PTA4, SRT50%, WRS65dB) and questionnaire (SSQ12) results before the pitch-matching session (Pre-fit) and 2 weeks later (Post-fit)

ID	Pre-fit PTA_4_ (dB)	Post-fit PTA_4_ (dB)	Pre-fit SRT_50%_ (dB)	Post-fit SRT_50%_ (dB)	Pre-fit WRS_65DB_ (%)	Post-fit WRS_65DB_ (%)	Pre-fit SSQ12	Pre-fit SSQ12
1	43.75	42.5	55	50	65	70	4.79	6.21
2	37.5	37.5	42.5	37.5	50	65	5.63	6.67
3	37.5	36.25	53	55	40	50	4.88	5.92
4	40	47.5	40	45	90	100	6.29	7.63
5	41.25	40	47	45	60	75	4.63	5.96
6	36.25	37.5	35	40	80	95	6.17	7.08
7	53.75	55	57.5	60	25	30	3.46	3.79
8	43.75	42.5	45	45	80	90	6.25	7.58
9	40	35	45	40	70	80	5.58	6.71
10	40	40	47.5	45	60	60	4.88	5.13
11	37.5	38.75	40	40	85	100	6.33	7.5
12	47.5	50	55	57.5	30	35	3.63	3.83
AVG	41.56	41.88	46.88	46.67	61.25	70.83	5.21	6.17
SD	5.03	6.07	7.04	7.41	21.44	23.92	1.00	1.33

For testing WRS the implanted ear received 65 dB speech while the contralateral ear received 60 dB noise

**Fig. 5:**
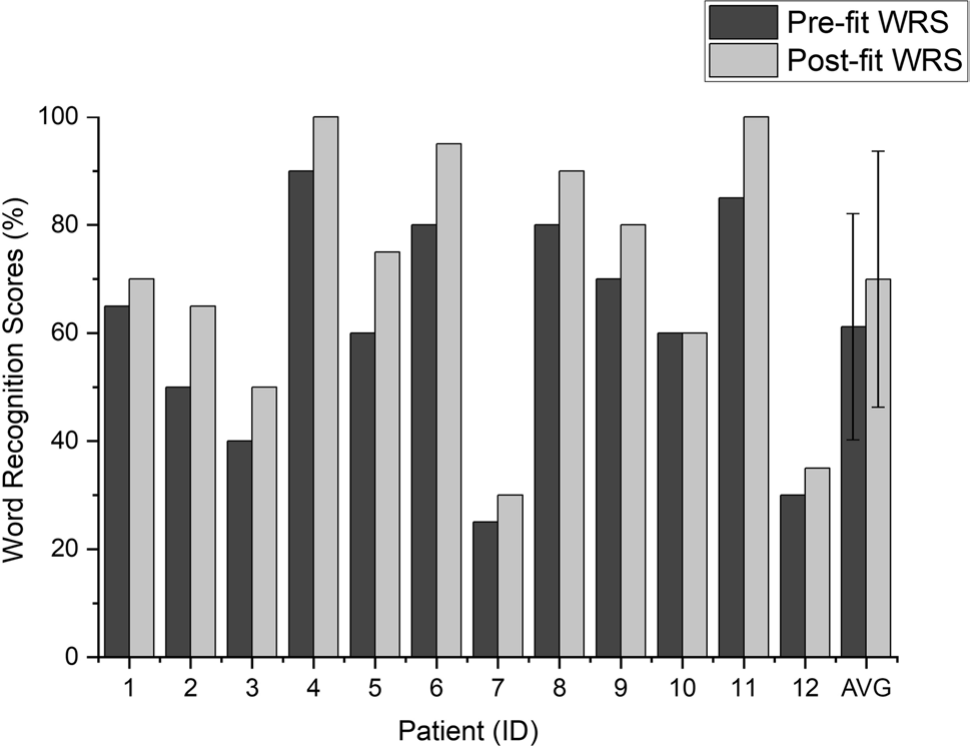
Word Recognition Score (WRS) results before the pitch-matching session (Pre-fit) and 2 weeks later (Post-fit). The implanted ear received 65 dB speech while the contralateral ear received 60 dB noise

**Fig. 6:**
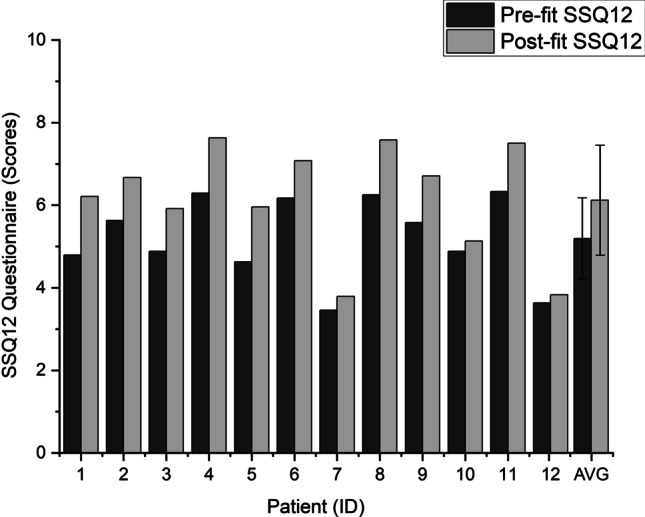
SSQ12 questionnaire results before the pitch-matching session (Pre-fit) and 2 weeks later (Post-fit)

The results of the SSQ12 questionnaire also showed significant improvement regarding speech intelligibility, sound localization, and sound quality (mean 0.96 points, SD 0.45 points, matched pairs *t *test comparison: *p < *0.001). Figures 5 and 6 display the visualizations of WRS_65dB_ and SSQ12 results of the 12 patients, respectively.

## Discussion

Although the improvement in speech comprehension and hearing quality was observable in nearly all individual patients, a notable heterogeneity of data appeared among the results.

Patients 4 and 11 already had high WRS_65dB_ and SSQ12 scores prior to the pitch-matching; however, they were not satisfied with the “unnatural” sound quality of the cochlear implant. In both cases, the CFs of the low-tone channels proved significantly mismatched, although the electrode array was fully inserted (impedances and NRT values were in the normal range, and the implanting surgeon reported full insertion in all participants). Following the reallocations of the frequency bands, the patients reported an improved hearing experience, and their audiological scores also increased. Patient 8 did not have hearing loss prior to the surgery (average of PTA_4_ < 25 dB HL in quiet), nevertheless, she was suffering from severe vertigo, therefore, underwent simultaneous labyrinthectomy with cochlear implantation. She quickly regained a satisfying speech comprehension after the surgery; however, she could not adapt to the different tone of the device for 2 years. With the new experimental FAT, as she described, “the sound of the speech processor was more comfortable and much more acceptable”. The results of Patients 7 and 12 showed less improvement, and they reported nearly no subjective change in their quality of hearing following the procedure. The explanation may likely be both patients were deaf for an extremely long period prior to the cochlear implantation (20 and 49 years, respectively), and as a result, they could only achieve very poor speech intelligibility with the CI. It is worth mentioning prior to surgery both patients had been informed, after a prolonged unaided period they could only expect the ability of detecting sounds and noises with the cochlear implant; however, it is unlikely they will be able to understand speech. Nevertheless, both patients insisted on undergoing the surgery. After the follow-up measurement, Patient 12 needed an additional fitting session to adjust the shifted T and C levels. Following the switch to the new FAT the WRS_65%_ scores of Patient 10 (the oldest participant, age 70) did not increase at all, yet he reported a “more natural color” of hearing. With the experimental FAT, some of the participants reported, they did not hear two different voices with their two ears anymore when someone was talking.

It is important to consider all the participants had been selected specifically for this experiment due to their unsatisfactory speech comprehension and their subjective general opinion regarding their quality of hearing (unable to adapt to the different tonal sonority of the CI). This may likely be the reason for the significant differences between the hearing intelligibility prior to and following the frequency matching session. Canfarotta compared postoperative CT scans of 111 cochlear implanted ears and found significant variability in frequency-to-place mismatch among CI-alone and Electric Acoustic Stimulation (EAS) users with default FAT parameters [35]. Mertens demonstrated the severity of the mismatch affects initial speech perception in noise; however, in general, the effect disappears following the first year of CI experience [36].

Tan concluded despite years of CI experience some electrode channels remain perceived as higher pitched than the acoustic frequencies with which they are associated [37]. In those special cases in which the patient is not able to adapt to the default FAT of the speech processor, it is beneficial to apply tonotopic pitch-matching. CT imaging may prove to be an effective tool for measuring frequency-place mismatch without requiring extensive psychophysical testing that are often subject to non-sensory bias [34, 38]. We concur if a patient has bilateral deafness and high-resolution postoperative CT imaging is accessible, measuring the Angular Insertion Depth (AID) is the preferable choice to determine the presumably best fitting frequency allocation. MED-EL’s Otoplan can be a useful tool for such optimization. Nevertheless, in SSD patients (where there are no significant functional anatomical differences between the two ears) we recommend the subjective pitch-matching method described in the current study to individualize the FAT.

It bears highlighting, although all participants had full-length insertion of the CI electrodes, none of the patients had postoperative CT scans or X-ray measurements to verify or disprove migration of the electrode in the cochlea prior to the pitch-matching session; therefore, it cannot be rejected as a possible explanation for the experienced frequency mismatch. Nevertheless, the telemetry showed no signs of incomplete insertion (e.g., elevated impedances, missing NRT responses), and all participants could differentiate between the pitches of all 22 stimulating channels.

It is important to state, there was no control group set to measure a potential Hawthorne Effect (HE), since all participants were informed about the idea of the experimental method.

## Conclusion

The results demonstrate the proposed pitch-matching method may increase the quality of hearing and speech comprehension in CI patients with SSD. This method can also usher in positive results in bimodal patients (cochlear implant in one side, hearing aid or bone conduction device on the other), or in pitch-matching the two CIs in bilateral patients. The disadvantages of the method include its time-consuming, and the patient needs to fully cooperate with the clinician and at a more demanding level. Understanding and performing the task of comparing pitches and not loudness levels is crucial to achieving beneficial results. The procedure, as an optional tool, can be implemented in any cochlear implant fitting software together with the automatization of the computations necessary to reallocate the frequency bands.

It will also be illustrative in a future study to compare the FATs generated with the two methods (image-guided vs. interaural pitch comparison-based reallocation) in consideration of patient satisfaction.

## Data Availability

All data approved by the participants of the study for publication and for online availability are provided within the paper. Table 2 and Table 3 contain the anonymized data of the individual patients. Upon special request, the authors can provide additional information regarding the settings of the speech processors of the individual participants.
